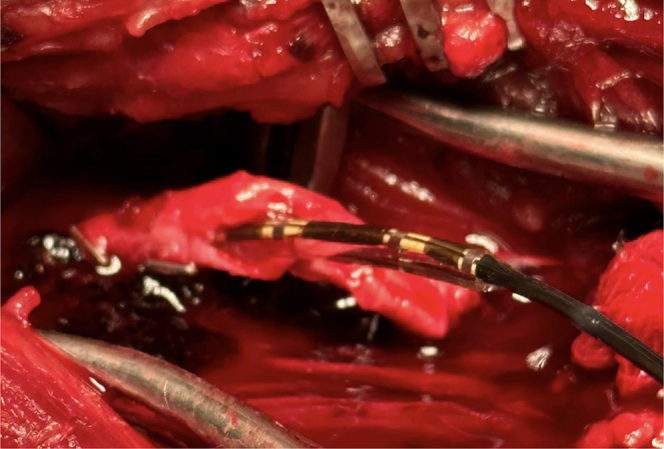# I only believe what I see: Cracking the code of calcification

**DOI:** 10.1016/j.jvscit.2024.101572

**Published:** 2024-07-11

**Authors:** Justine Longchamp, Rafael Trunfio, Céline Deslarzes-Dubuis

**Affiliations:** Department of Vascular Surgery, Centre Hospitalier Universitaire Vaudois and University of Lausanne, Lausanne, Switzerland

Intravascular lithotripsy (IVL) represents an innovative technique for remodeling calcified plaques. This technique has shown promising results in the management of peripheral artery disease.^1^, ^2^, ^3^ This report describes a successful case of IVL-assisted distal bypass.

## Case report

A 81-year-old man was referred for incapacitating foot pain associated with toe necrosis (Rutheford 5 stage, Wound, Ischemia, foot Infection stage 4). The duplex ultrasound examination showed femoro-popliteal occlusion, monophasic flow in the anterior tibial artery (ATA), both peroneal and tibial posterior arteries were occluded (GLASS stage III). Laser toe plethysmography showed no oscillation. The computed tomography angiography highlighted highly calcified below-the knee arteries (*A*). The patient was consented for a composite (contralateral great saphenous vein anastomosed with cryopreserved allograft distally) femoro-ATA bypass and provided consent to this publication. Proximal anastomosis was performed on the common femoral artery. ATA was dissected over 15 cm and was found to be highly calcified, looking like an incompressible pipe. Longitudinal arteriotomy with endarterectomy were performed. Circumferential calcified plaque was not ending on distal ATA. Distal plaque fixation was not feasible even with a calcium-dedicated needle.

We decided to perform ATA IVL to soften the plaque. First, a 0.014 Spartacore (Abbott Vascular, Abbott, IL) wire was inserted a few centimeters trough ATA. Then, a 3 × 40-mm Shockwave S^4^ (Shockwave Medical, Santa Clara, CA) balloon was placed (*B*/Cover image) and inflated according to the instructions for use.^4^ Eight cycles of 20 pulses were performed while moving slightly the balloon to focus the pulses at the distal anastomosis spot. By the end of IVL, the artery was softer macroscopically, and the distal anastomosis was performed using Prolene (Ethicon) 6-0. No anastomosis leak was seen, final angiography was done to evaluate distal anastomosis and run off (*C*). Therapeutic anticoagulation with heparin was started 4 hours after the surgery together with aspirin. The patient had no more foot pain, and duplex ultrasound examination at postoperative day 4 showed triphasic flow in the ATA (*D*); the toe-brachial index was 0.71.

## Conclusions

To our knowledge, this description of IVL is the first use to help soften the plaque of a highly circumferentially below-the-knee artery, allowing the completion of an anastomosis.

**Figure:**
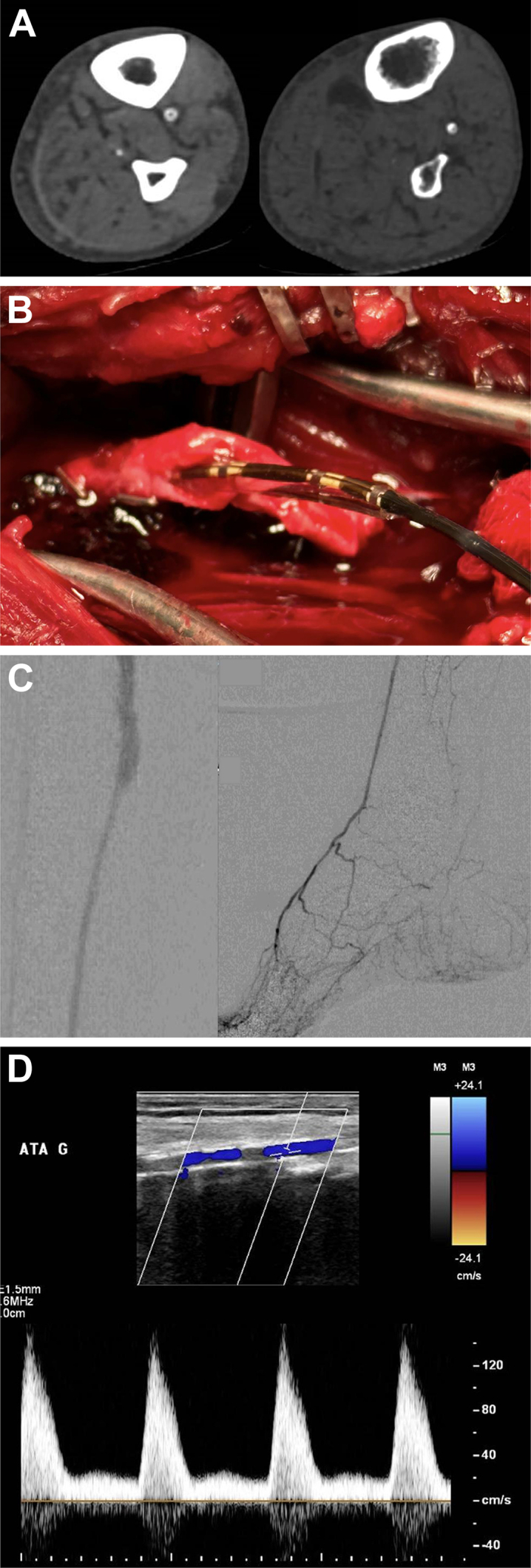

## Disclosures

None.

## Figures and Tables

**Cover image:**